# Work-related Musculoskeletal Disorders in Iranian Office Workers: Prevalence and Risk Factors

**DOI:** 10.25122/jml-2018-0054

**Published:** 2018

**Authors:** Fariborz Mohammadipour, Mohammad Pourranjbar, Sasan Naderi, Forouzan Rafie

**Affiliations:** 1. Department of Biomechanics, Faculty of Physical Education and Sport Sciences, Shahid Bahonar University of Kerman, Kerman, Iran; 2. Neuroscience Research Center, Kerman University of Medical Sciences, Kerman, Iran

**Keywords:** Musculoskeletal disorder, RULA, ROSA, office workers

## Abstract

**Objective:** This study aimed to identify the prevalence of musculoskeletal disorders (MSDs) and ergonomic risks for Kerman University of Medical Sciences’ office workers.

**Methods:** The study sample comprised all office workers in the University and the sample included 129 women and 121 men. Data on MSDs were derived from the Nordic Musculoskeletal Questionnaire, while ergonomic data were collected through two direct observations via the rapid upper limb assessment (RULA) and the rapid office strain assessment (ROSA) method.

**Results:** The results showed that the highest prevalence rates of MSDs were in the lower back (72.4%) and neck (55.2). Results of the postural assessment revealed that 68.8% of the participants’ require “further investigation in order to modify their posture” and 27.6% need to “modify their posture soon.” From the workstation analysis, the majority of the office workers were at a medium (55.2%) and high-risk level (27.6%). Results also revealed a significant association between some of MSDs in the lower back and neck with the RULA and ROSA score.

**Conclusions:** Based on the results, for the prevention of MSDs, there should be ergonomics workshops for workers to be aware of ergonomics factors in the office. The ergonomics training must also be used in offices; the design of workstations should be improved.

## Introduction

The design and maintenance of a suitable work environment are one of the objectives of Ergonomics to improve the worker’s performance, reduce stress and fatigue at work. Application of ergonomics is very significant in the area where manual activities directly affect the physical and mental health of the employee [1]. In ergonomics, the posture and movement of a worker are important information for determining the risk of musculoskeletal disorders (MSDs) in the workplace [2]. MSDs have been common complaints among workers involved in static work or tasks requiring the repetitive motion of the upper limbs and prolonged computer work. Office workers are the one group which may impact on chronic musculoskeletal health problems. Office work represents a complex physical work environment, with interactions among the various dimensions of the workstation, equipment and job content. Recent research reviews have confirmed the dose-response association between the number of hours working at a computer workstation and the risk of MSDs which include pain and other symptoms in the shoulder-neck, back and upper limbs particularly [3–7]. The prevalence of MSDs among persons with frequent computer use (3–5 h a day) ranges from 40% among college students [8], 50% among new workers in the first year on the job [9] to over 70% of university staff and students [10].

In Choobineh, Daneshmandi and Tabatabaee’s study, 2934 female employees from 15 Iranian workplace settings distributed throughout the country were analyzed. The study revealed high prevalence rates of WMSDs in different body regions among female workers in Iran and the highest prevalence rate of WMSDs symptoms, in descending order, was related to lower back, shoulders and wrists/hands, respectively. WMSDs among the sample workers were found to be associated with age, job tenure, daily working hours, type of activity and working schedule [11]. In another study, Alavi, Abbasi and Mehrdad selected 1630 Iranian office workers via a random multistage cluster sampling method, of which 1488 completed the tasks of the study. They concluded that MSDs had a high prevalence in their case and various risk factors in the workplace may contribute to MSDs in different upper extremities [12]. The results of Mozafari et al.’s study showed that the musculoskeletal issues had a high frequency among office workers (55.5% of office workers had musculoskeletal disorders in one year). They reported that the most common symptoms over the course of one year were in the knee (36.4%) and lumbar regions(12.1%) in the case of office workers. Musculoskeletal disorders showed statistically significant association with work duration, age, and BMI. Office workers usually remain in prolonged uncomfortable postures and high static muscle load which may imply a risk for development of problems [13].

The prevention of MSDs among office workers depends on accurate identification of exposure to occupational risks. Different methods and tools have been developed to assess exposure to risk factors for work-related MSDs. They can be divided into three groups according to the measurement technique. They are the self-report, direct measurement and observational methods [11, 12]. Observational methods consist of directly observing the worker and the corresponding tasks, such as Rapid Upper Limbs Assessment (RULA) [13], the Rapid Entire Body Assessment (REBA) [14] and the Rapid Office Strain Assessment (ROSA) [15]. Among all the techniques, RULA and ROSA are reliable and valid for evaluating the computer workstation and other types of posture which involve the upper limbs. Therefore, the aim of this study was to investigate the prevalence of MSDs and assess ergonomics risk for MSDs by analysis of body posture and workstation in a work environment among office workers.

## Method

### Participants

Study participants were employees at the University of Medical Sciences in Kerman, Iran. 250 office workers (including 129 women and 121 men), with ages ranging between 25 to 55 years, weighing approximately 68.70 ± 8.50 kg (57–97 kg), and heights of 163.21 ± 4.12 cm (150–186 cm), participated in the survey. Participants worked an average of at least 7 h per day, five days a week at an office computer station and had been employed in this position for at least a year. The participants signed an informed consent form, and the study’s procedures were approved by the Institutional Review Board for the protection of human subjects in both experimental sites.

### Procedure

In this study, the data were obtained with questionnaires and by direct observation. In order to determine the prevalence of MSDs in different limbs of the workers, the Nordic questionnaire was used [16]. The questionnaire enquired about the history of the experience of MSDs in nine body sites (neck, shoulders, elbows, wrists/hands, upper back, lower back, hips/thighs, knees and ankles/feet) over the past weeks and over the past year. Ergonomic risk factors were assessed through direct observation of the subject’s postures and workstation equipment at their workstations by means of the RULA and ROSA tools [13].

The RULA measure for computer users which quantifies the grade of the musculoskeletal risk of the sitting posture on a 1–7 scale was used to analyse the posture of the body. Higher RULA scores indicate greater levels of risk factors causing load on the structures of body segments. The grade is calculated based on the degree of angles between various body segments and their recommended postures according to criteria derived through interpretation of previous relevant studies. In addition to these data, ROSA was used to evaluate the workstations suitability, measuring indices relating to the workstation (chair height, seat pan depth, armrest, and back support), computer (monitor, mouse, and keyboard), telephone and duration of time spent in each posture or activity [15].

The ROSA method has been designed to quantify the risks associated with computer work quickly and to establish a course of action to characterize the level of risk of the workplace and to discover the postures that workers adopt at the workplace. ROSA final scores ranged in magnitude from 1 to 10, with each successive score representing an increased presence of risk factors. According to ROSA, there were three risk levels: low, medium and high. Ergonomics risk levels showed that ROSA scores over 5 points were at least high-risk level concluding that the workplace should be improved and strictly assessed.

### Statistical analysis

Descriptive statistics of the general characteristics, work, and workplace characteristics and ergonomic risks of the study population were presented as numbers, percentages, and mean ± standard deviation. In order to understand which ergonomic risk factors with RULA and ROSA relate to MSDs, chi-square test and Pearson correlation coefficients were used. The factors included were RULA scores, ROSA scores and pain score of various body parts. SPSS version 23 was used for all statistical analyses, with significance set at p< 0.05.

**Table 1:** Nordic questionnaire results (n=250)

Area of body affected	Occurrence in last 12 months No. (%)
Neck	138 (55.2)
Shoulders	129 (51.6)
Elbows	17 (6.8)
Wrists/Hands	60 (24.0)
Upper back	68 (27.2)
Lower back	181 (72.4)
Hip/Thigh	60 (24.0)
Knee	112 (44.8)
Ankle/Feet	26 (10.4)

**Table 2:** Result of RULA* final score (n=250)

Action level	No. (%)
Action level 1	9 (3.6%)
Action level 2	172 (68.8%)
Action level 3	69 (27.6%)
Action level 4	0 (0%)

* RULA= Rapid upper limb scale

**Table 3:** Result of ROSA* final score

Risk level	No. (%)
Level 1: low risk	43 (17.2%)
Level 2: medium risk	138 (55.2%)
Level 3: high risk	69 (27.6%)

* ROSA= Rapid Office Strain Assessment

**Table 4:** Relationship between RULA risk factors and musculoskeletal disorders

Body area	C	D	Final score
Neck	r= 0.146	r= 0.399	r= 0.360
	P= 0.448	P= 0.032*	P= 0.055
Shoulders	r= 0.507	r= −0.064	r= 0.310
	P= 0.005*	P= 0.741	P= 0.102
Elbows	r= 0.275	r= 0.132	r= 0.177
	P= 0.149	P= 0.494	P= 0.358
Wrists/Hands	r= 0.295	r= −0.104	r= 0.142
	P= 0.120	P= 0.590	P= 0.463
Upper back	r= 0.028	r= 0.272	r= 0.239
	P= 0.885	P= 0.153	P= 0.211
Lower back	r= 0.340	r= 0.453	r= 0.492
	P= 0.071	P= 0.014*	P= 0.007*
Hip/Thigh	r= −0.053	r= 0.274	r= 0.142
	P= 0.786	P= 0.150	P= 0.463
Knees	r= −0.048	r= 0.132	r= 0.176
	P= 0.804	P= 0.495	P= 0.362
Ankles/Feet	r= 0.032	r= 0.205	r= 0.215
	P= 0.869	P= 0.285	P= 0.263

RULA= Rapid upper limb scale C score= Score of arms, forearms and wrists postures + muscle use + force D score= Score of neck, trunk and lower extremity postures + muscle use + force

* Indicates a significant correlation

## Results

### Prevalence

The highest prevalence rate of MSDs was in the lower back (72.4%), neck (55.2%) and shoulders area (51.6%) and the lowest prevalence rate of MSDs was in elbows (6.8%) and ankle area (10.4%). The findings from this study also showed that 88.4% of the subjects had experienced MSDs over the last 12 months. Table 1 presents the results of the Nordic questionnaire. The result of the postural analysis, obtained through the RULA approach, showed that 3.6% of participants were under action level 1, which indicates posture is acceptable if it is not maintained or repeated for prolonged periods. 68.8% of participants were under action level 2, which requires further investigation and changes may be required and 27.6% of participants were under action level 3, which indicates that changes are needed soon (Table 2). Results of the evaluation of the workstations through the ROSA approach revealed that most workstations (82.8%) were at a medium and high level of risk, which indicates workstations should be assessed further (Table 3).

### Correlation

Table 4 presents the association between MSDs with RULA *C* score (score of arms, forearms, and wrists postures + muscle use + force), RULA *D* score (score of the neck, trunk, and legs postures + muscle use + force) and RULA final score. Correlation Analysis showed that MSD in the neck area was significantly correlated with the RULA *D* score (r=0.399, P=0.032), MSD in shoulders area was significantly correlated with RULA *C* score (r=0.507, P=0.005) and MSD in lower back area was significantly correlated with RULA *D* score (r=0.453, P=0.014) and RULA final score (r=0.492, P=0.007).

Table 5 presents the association between MSDs with chair score, monitor and telephone score, mouse and keyboard score and ROSA final score. Correlation Analysis showed that MSD in the neck area was significantly correlated with monitor and telephone score (r=0.375, P=0.048), MSD in upper back area was significantly correlated with monitor and telephone score (r=0.435, P=0.018) and MSD in lower back area was significantly correlated with chair (r=0.370, P=0.048), monitor and telephone score (r=0.410, P=0.027) and ROSA final score (r=0.412, P=0.026). Also, there was a significant correlation between the ROSA score and RULA posture score (r=0.565, P=0.001).

**Table 5:** Relationship between ROSA risk factors and musculoskeletal disorders

Body area	Chair	Monitor and Telephone	Mouse and Keyboard	Final score
Neck	r= 0.097	r= 0.375	r= 0.033	r= 0.162
	P= 0.618	P= 0.048*	P= 0.863	P= 0.400
Shoulders	r= 0.195	r= 0.014	r= 0.306	r= 0.260
	P= 0.312	P= 0.944	P= 0.106	P= 0.174
Elbows	r= 0.010	r= −0.185	r= 0.179	r= 0.089
	P= 0.958	P= 0.338	P= 0.354	P= 0.648
Wrists/Hands	r= 0.032	r= 0.108	r= 0.262	r= 0.076
	P= 0.869	P= 0.576	P= 0.170	P= 0.694
Upper back	r= 0.221	r= 0.435	r= 0.106	r= 0.274
	P= 0.248	P= 0.018*	P= 0.584	P= 0.151
Lower back	r= 0.370	r= 0.410	r= 0.195	r= 0.412
	P= 0.048*	P= 0.027*	P= 0.312	P= 0.026*
Hip/Thigh	r= 0.326	r= 0.268	r= 0.227	r= 0.309
	P= 0.084	P= 0.160	P= 0.237	P= 0.103
Knee	r= 0.248	r= 0.185	r= 0.153	r= 0.176
	P= 0.195	P= 0.336	P= 0.428	P= 0.362
Ankle/Feet	r= 0.109	r= 0.091	r= 0.046	r= 0.103
	P= 0.572	P= 0.638	P= 0.811	P= 0.594

ROSA= Rapid Office Strain Assessment

* Indicates a significant correlation

## Discussion

This study was conducted to identify the prevalence of MSDs and ergonomic risks in office workers at Kerman University of Medical Sciences. There is limited literature on assessment and improvement of ergonomic conditions in Iranian workers’ workstations. To help fill this gap, the present study surveyed compliance of workstations and postures of the employees to the computer-related ergonomic standards in Iranian office workers. The results of the current study showed that these workers had both a high level of MSDs as well as high ergonomic risks. In this study, 88.4% of employees experienced MSDs at least in one limb due to poor posture imposed by their workstation conditions. Studies revealed that awkward posture leads to the development of MSDs [2, 9]. In the current study, the working posture of the university staff most of the cases was at Action level 2 and 3 which indicate the changes may be required. This was an expected result due to the significant percentage of the office workers’ existing MSDs. The high RULA scores may be related to workstation design. ROSA-based results showed that more than 82% of workstations are not ergonomic and some previous studies have also reported the non-ergonomic workstations as the most frequent work-related risk factor of MSDs [17]. 

It was shown that training of ergonomic principle and practice alone, without redesign of workstation layout, cannot significantly reduce the ergonomic risk factors and thereby the related MSDs [18]. Regarding the ROSA assessment, the scores assigned to the monitor are related to the positioning of the head in relation to the screen, where workers are often facing very low screens, forcing a flexion of the cervical spine, as well as many workplaces, do not present a documents support, which forces office workers to flex and/or rotate the neck to analyze papers placed on the side of the desk.

 The constant movements of flexion or rotation of the cervical spine can cause discomfort in the head and shoulders. At the level of the neck and in the trapezius muscle region, it is known that there is a positive relationship between the flexion of the neck and MSDs in this region as well as in the lumbar spine, whose combined movement of flexion and rotation of the trunk and forced movements are related to the presence of discomfort [2]. As for the phone, most workplace stations did not have available headsets, which often forces workers to hold the phone between their head and shoulder causing a considerable strain on the spine and shoulder musculature [19]. Based on these assessments, in the current study, the score of the monitor and telephone was significantly positively associated with the neck, upper back, and lower back symptoms.

The scores obtained from chair analysis showed that many employees did not use standard chairs. Also, the assessments revealed that many workers did not use the armrests, the arms being supported by the desk which often causes elevation of shoulders and, consequently, the increase of tension in the muscles of the neck and shoulders. However, some studies demonstrated that supporting the forearm on the work surface may increase comfort and decrease the muscular load of the neck and shoulder [20, 21]. 

As for lumbar support, some workers did not present chair support for the lumbar spine, with their trunk in flexion, which increases muscle activity in this region. Maintaining a sitting posture for a long time in static positions causes some MSDs [22, 23]. The results of this study also showed that RULA D score and ROSA chair score were significantly positively related with lower back symptoms. In this study, two variables (body posture and workstation) were determined as significant predictors of the likelihood of the upper limb MSDs. A higher prevalence of lower back, neck, and shoulders MSDs in office workers was caused by bad body posture and inappropriate workstations. Therefore, employees’ postures at their workstations needed to be investigated and some changes were required immediately. It is essential that ergonomic programs for the workers in the study population be put into action immediately and medical treatment for those with high symptomatic and risk levels be provided. Our suggestions for future studies include associations between demographic data and complaints and examinations of risk assessments.

## Conclusion

Although the effect of etiological mechanisms on causing MSDs is still poorly understood, studies have provided evidence that environmental and personal factors influence the occurrence of MSDs. The results of the current study confirmed some of these relationships. The office workers participating in this research were found to have a high level of both MSDs and ergonomic risks. These findings can be used to guide MSD prevention efforts for office workers in Iran.

## Conflict of Interest

The authors confirm that there are no conflicts of interest.
